# GrAfSS: a webserver for substructure similarity searching and comparisons in the structures of proteins and RNA

**DOI:** 10.1093/nar/gkac402

**Published:** 2022-05-25

**Authors:** Nur Syatila Ab Ghani, Reeki Emrizal, Sabrina Mohamed Moffit, Hazrina Yusof Hamdani, Effirul Ikhwan Ramlan, Mohd Firdaus-Raih

**Affiliations:** Institute of Systems Biology, Universiti Kebangsaan Malaysia, 43600 UKM Bangi, Selangor, Malaysia; Department of Applied Physics, Faculty of Science and Technology, Universiti Kebangsaan Malaysia, 43600 UKM Bangi, Selangor, Malaysia; Department of Applied Physics, Faculty of Science and Technology, Universiti Kebangsaan Malaysia, 43600 UKM Bangi, Selangor, Malaysia; Advanced Medical and Dental Institute, Universiti Sains Malaysia, Bertam, Kepala Batas 13200, Pulau Pinang, Malaysia; School of Medicine, University College Dublin, Belfield, Dublin 4, Ireland; Institute of Systems Biology, Universiti Kebangsaan Malaysia, 43600 UKM Bangi, Selangor, Malaysia; Department of Applied Physics, Faculty of Science and Technology, Universiti Kebangsaan Malaysia, 43600 UKM Bangi, Selangor, Malaysia

## Abstract

The GrAfSS (Graph theoretical Applications for Substructure Searching) webserver is a platform to search for three-dimensional substructures of: (i) amino acid side chains in protein structures; and (ii) base arrangements in RNA structures. The webserver interfaces the functions of five different graph theoretical algorithms – ASSAM, SPRITE, IMAAAGINE, NASSAM and COGNAC – into a single substructure searching suite. Users will be able to identify whether a three-dimensional (3D) arrangement of interest, such as a ligand binding site or 3D motif, observed in a protein or RNA structure can be found in other structures available in the Protein Data Bank (PDB). The webserver also allows users to determine whether a protein or RNA structure of interest contains substructural arrangements that are similar to known motifs or 3D arrangements. These capabilities allow for the functional annotation of new structures that were either experimentally determined or computationally generated (such as the coordinates generated by AlphaFold2) and can provide further insights into the diversity or conservation of functional mechanisms of structures in the PDB. The computed substructural superpositions are visualized using integrated NGL viewers. The GrAfSS server is available at http://mfrlab.org/grafss/.

## Graphical Abstract

**Graphical Abstract:**
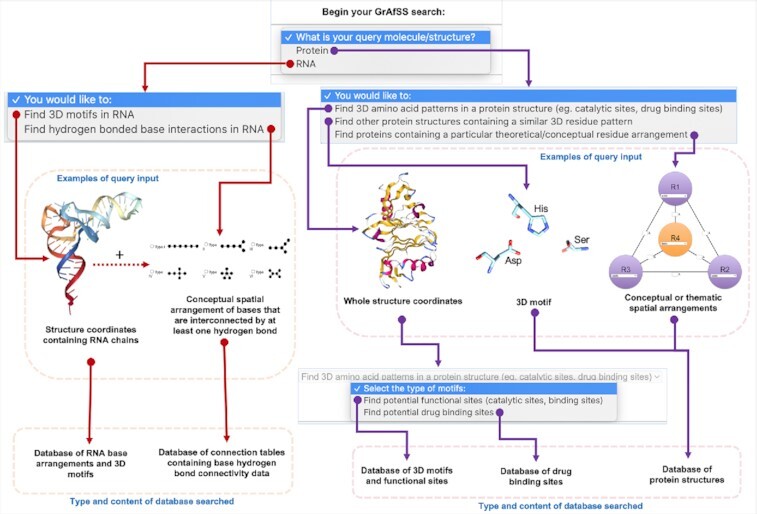
The GrafSS server enables searches for 3D arrangements of amino acid side chains or 3D arrangements of RNA bases in PDB or mmCIF structure coordinate files.

## INTRODUCTION

For the first thirty years of its existence (1971–2001) (1), the Protein Data Bank (PDB) (2,3) registered only 16,401 entries of structure coordinate data. After that point, especially within the past decade, improvements in high-throughput approaches and structure determination methods as well as a larger structural biology community have helped contribute to a more rapid increase in the number of deposited structures. In its 50^th^ year, a total of 185,533 coordinates were available and this number is likely to exceed the 200,000 threshold by 2023. The number of RNA structures, which had always lagged behind that of proteins, has also seen a similar trend (4). Recent improvements in the accuracy of protein structure prediction methods using neural networks such as RosettaFold (5) and AlphaFold2 (6) have resulted in over 360,000 coordinate files for the proteomes of model organisms and pathogens (7). Comparing the different structures of proteins and RNA available in the PDB for similarities or differences at three-dimensional (3D) level is a useful means of gleaning functional insights, including mechanistic variations at the atomic level.

Structure similarity searching has generally focused on whether protein structures contain similar folds even when their sequences have diverged up to the point of having no detectable sequence similarities. One established tool used for such a purpose is DALI (8). Typically, a newly solved structure, especially one that does not share any detectable sequence similarity to examples in the PDB, would be subjected to a fold similarity search such as DALI. However, should DALI not be able to provide any potential linkage to known functions by way of fold similarity, it would then be useful to identify whether there exists any similarities to functional substructures such as catalytic sites, ligand binding sites and other interfacing residues. In this work, a substructure refers to either: (i) a specific 3D arrangement of amino acid residues; (ii) a specific 3D arrangement of RNA bases; or (iii) a specific cluster of RNA bases that are interconnected by hydrogen bonds. Several tools are available to search for substructural similarities in proteins and RNA. This is the premise behind the functions and features for the webserver that we report in this paper.

The capacity to search for similar amino acid 3D arrangements via a web browser interface is provided by servers such as ASSAM, SPRITE (9), ProBis (10) and RASMOT-3D PRO (11). For RNA chain containing structures, a similar substructure search function for annotating RNA base 3D arrangements is available via web servers such as MC-Annotate, WebFR3D, NASSAM, COGNAC, and ClaRNA (12–16). However, web server access only solutions may have limitations in terms of high-throughput processing capacity and programmatic access. For example the work reported for compiling the database of 3D sites that are similar to known drug binding sites for the purpose of drug repositioning (17,18) and the work reporting the discovery of novel base triples in RNA structures (19,20) were carried out by standalone versions of the ASSAM and NASSAM computer programs and not their respective web services. Stand-alone tools that are able to carry out high-throughput analysis fill a complementary gap in the scenarios presented above. They can also be important components in a pipeline that provide the desired functionalities required of the web services such as database generation. One example of a stand-alone software that is highly similar to the webservers for protein substructure comparison is the Graph-based Local Structure Alignment (G-LoSA) program which utilize the chemical features of the amino acids and carries out iterative maximum clique searching and fragment superposition for local substructure alignments (21). For RNA substructure searching, tools such as RNAMotifScan, CompAnnotate, Local STAR3D, and LCS-TA (22–25) provide a similar stand-alone and high-throughput processing capability.

It is clear that although there are many different similarly intended algorithms for substructure searching, the approach, accessibility and processing capacity (including input and output formats) are different thus making the available programs complementary to each other and can be integrated into a specific pipeline to provide a more comprehensive analysis. For example, only ASSAM and SPRITE provide a search capacity that utilize 3D superpositions of the side chains while other tools generally use the C-alpha positions of the amino acid residues for substructure comparisons. As a consequence, ASSAM and SPRITE searches may miss occurrences where the C-alphas overlap and the side chains do not. However, the use of graph theoretical algorithms appear to be a common approach for the different protein substructure searching tools available despite the differences in how the components being searched for are represented and processed.

For RNA substructure searches, all the webservers we benchmarked against as part of this work adopt a geometrical approach to compare the spatial arrangements of RNA bases. However, only MC-Annotate, NASSAM and COGNAC utilize graph theoretical algorithms to solve the sub-graph problem. As was observed for the protein substructure searching tools, each RNA substructure searching algorithm use different representations as the graph's nodes and edges. The stand-alone RNA substructure search applications use pairwise structural alignment based approaches to identify motifs. For example, LCS-TA utilise the torsion angles of the RNA backbone to execute a computationally non-demanding divide and conquer technique to identify local substructure similarity thus making the method quite distinct compared to the base-centric geometric approach adopted by other algorithms such as MC-Annotate, NASSAM and COGNAC.

The variety of approaches offered by the currently available tools for substructure searching create a rather complementary protein and RNA substructural analysis ecosystem that can adequately cater to the needs of a wide userbase that require different metadata, have different biological questions and different functional context requirements. This allows for a newly solved structure or a new computationally generated one with no detectable sequence or fold similarity to available PDB records to be submitted for a 3D substructure search to identify sites associated with specific mechanisms or interactions. Alternatively, the results of expert visual examinations of such new structures can identify potential functional sites that can in turn be used as a query to identify similar arrangements in other PDB structures. The substructural searching capability we present and discuss here can extend the search for functional similarity beyond the sequence and fold similarity options that are widely practiced.

## MATERIALS AND METHODS

### The GrAfSS webserver interface

**Figure 1.**
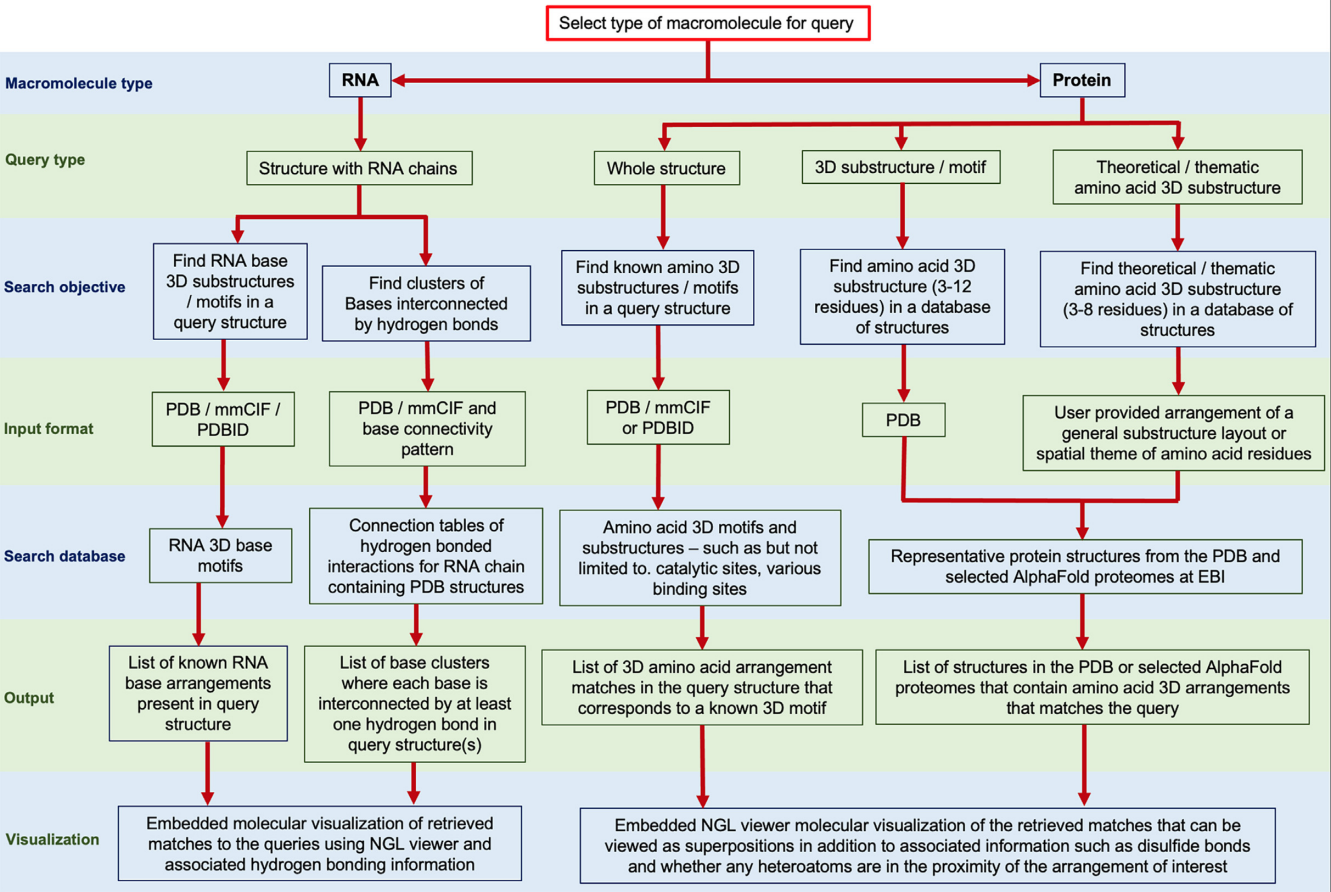
An overview of the options and flow for a GrAfSS search that begins with selecting the type of macromolecule and progresses to the different search programs and database options based on the user's intended search objectives. The different query formats and types of databases searched are presented to better illustrate the different searches that the GrAfSS webserver can execute.

The GrAfSS webserver integrates the functions of five established algorithms – ASSAM, SPRITE, IMAAAGINE, NASSAM, COGNAC (9,14,15,17,26) – that have been upgraded and merged into a single one-stop substructure similarity searching suite. The GrAfSS interface was made as simple as possible in order to guide users from diverse backgrounds into selecting the most suitable program and database combination for their search requirements (Figure 1). Previously, users were required to decide from the literature which substructure searching program was most suitable for their analysis. This was due to the fact that the five search programs were released at different times over a period spanning several years and were thus disconnected. As the user base for the servers increased, we revised the search and results examination functions for each program in addition to carrying out substantial updates to the databases that we present as the GrAfSS webserver.

The webserver interface and results pages were developed using PHP and Python. GrAfSS users are first presented with an option of whether their queries are structure coordinates of proteins or RNA (containing RNA chains). Once the type of macromolecule for searching has been determined, users are then provided options related to the objectives of their search (Figure 1). The molecular visualization capability in the previous standalone servers was provided through Jmol and required the installation of additional plug-ins. All embedded molecular viewers within the GrAfSS suite have been migrated to the WebGL NGL tool which provide users with better graphics rendering features and more integrated analysis tools without the requirement of additional plug-ins. The embedded NGL molecular viewers (27) allow users the option of visualizing the substructure matches, and where relevant, they are also able to visually examine how well the query superposes to the retrieved hit.

Due to space limitations, users are expected to save their search results before administrative deletions of output files; this can be done either via downloading: the raw text outputs, a csv file, or a PDF file as per the instructions provided. The csv and PDF files retain their connectivity to the server's NGL viewers thus allowing users to still view the matches for runs that have been removed from the server's storage. These new features were not available in the previous standalone webservers which mainly provided search results as tables displaying hits ranked according to the RMSD values. Changes that have been made to the processing workflows and databases have also led to faster computation times without affecting the precision and recall values reported for the original algorithms.

### Search algorithms

**Figure 2.**
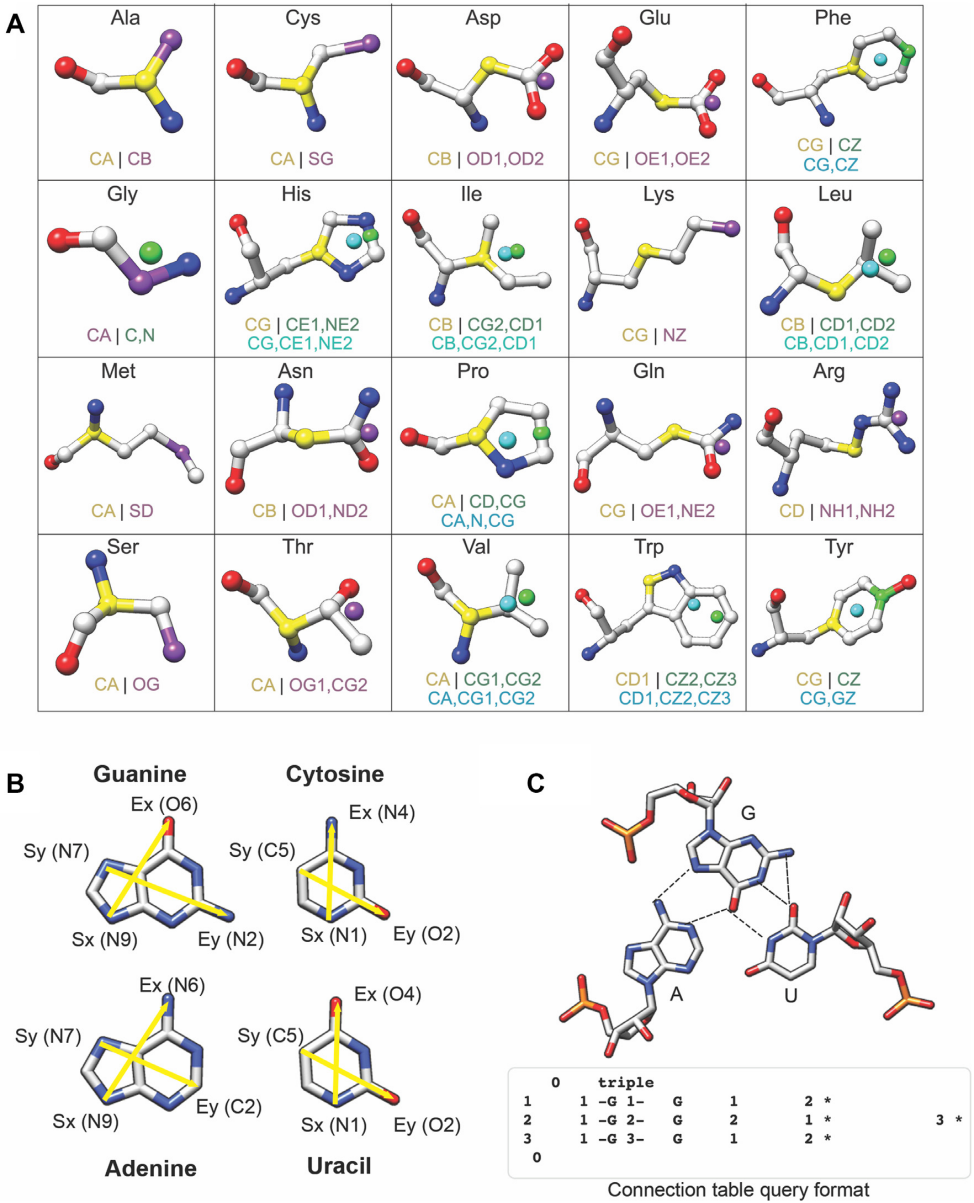
Graph theoretical representations of amino acid side chains and RNA bases used in GrAfSS. (**A**) The 20 amino acids are represented by Key Start (indicated in yellow) and Key End (indicated in green) pseudo-atoms as graph nodes for the SPRITE and ASSAM algorithms; for the IMAAAGINE algorithm, a single Key pseudo-atom is used as indicated in cyan. The overlapping Key Start/End and single Key pseudo-atom for both algorithms are indicated in purple. (**B**) The four RNA bases are represented by pseudo-atom vectors that are also the nodes of a graph. (**C**) The connectivity of the bases by hydrogen bonds (dotted lines) are represented in a connection table (lower panel).

The computational processes executed by the GrAfSS server are well established. The GrAfSS server executes searches for substructural similarities using graph theoretical representations of the 3D structures of proteins and RNA. For protein substructural similarity searching, the side chains of amino acids are represented by different pseudo-atom vectors for each of the twenty common amino acids (Figure 2A). These pseudo-atoms form the nodes of a graph while the distances between the pseudo-atoms form the edges thus allowing for the spatial relationships between the side chains to be represented. The use of 3D side chain arrangement similarities has an advantage over methods that only match the C-alpha positions because there are many examples where the side chains overlap even when the C-alpha positions do not (9). NASSAM works in a similar way with the difference being the graph's nodes are pseudo-atom representations for the RNA base residues (Figure 2B).

The SPRITE, IMAAAGINE and NASSAM programs compute substructural similarities by utilizing the Ullmann subgraph isomorphism algorithm (28). The ASSAM program implements the Bron and Kerbosch maximal clique searching algorithm to search for substructures in a database of protein structures (29). Since the 3D arrangements are not expected to be exactly the same, a distance tolerance is incorporated into the searches. The default value for amino acids is 1.5Å while a 30% value is used in the RNA searches. These values were used after extensive testing revealed them to provide an optimal balance of precision and recall. Some of the GrAfSS query options allow for these values to be changed in order to widen the search. The COGNAC searches differ from the others because the bases must be connected by hydrogen bonds to be considered a substructure (Figure 2C upper panel) and thus does not use the distances between the nodes as the graph's edges. In this case, the hydrogen bonding information are collected in connection tables (Figure 2C lower panel) where the subgraph isomorphism is again computed using the Ullmann algorithm (28).

### Datasets and input formats

The databases that the SPRITE, ASSAM, IMAAAGINE and NASSAM programs search against are in the same pseudo-atom vector representations of PDB or mmCIF formatted files as used for the queries, while the COGNAC searches utilise a database of connection tables containing information on the hydrogen bond connections between the bases. The user inputs can be provided either in the PDB and mmCIF format or as a substructural arrangement schema. These inputs are automatically converted into the same formats as the search databases without any further user intervention (Table 1). Searches for the presence of known amino acid side chain arrangements in structures already available in the PDB can also be carried out using a PDBID. Three types of search objectives are available to the user: (i) determining whether a structure has known motifs or 3D arrangements, (ii) determining whether a motif or 3D arrangement of interest is present in other structures and (iii) determining whether a cluster of hydrogen bonded base interactions is present in a search database or reference structure (Table 1). Two sources of structure coordinate data are used as the databases - the PDB (http://www.rcsb.org/pdb) and the AlphaFold protein structure database (https://alphafold.ebi.ac.uk/). For the NASSAM and COGNAC programs, additional hydrogen bonding data are generated by an in house program, HBPRED, using parameters as previously reported by Firdaus-Raih *et al.* (15).

**Table 1.** Information on the corresponding input formats, program, example search objectives and the source datasets for the databases used

	Program	Search objective	Query (format)	Data set source for search database
Protein	SPRITE	Search for the presence of a 3D substructure composed of amino acid side chain arrangements in a protein structure.	Protein structure coordinate file (*.pdb, *.cif) or a four character PDBID.	•Catalytic Site Atlas (34), • 3D-Footprint (35), • ProCarb (36) and • Substructures and motifs that were curated from literature or specific interactions, such as the interfaces of protein-drug complexes (17).
	ASSAM	Search for protein structures having a similar 3D substructure as the query.	3D motif or substructure composed of 3–12 residues (*.pdb).	• Non-redundant PDB datasets at 30% and 35% sequence identity excluding mutant structures; • Non-redundant PDB datasets at 30% and 35% sequence identity including mutant structures;
	IMAAAGINE	Search for protein structures having a similar 3D substructure as the query.	Conceptual / hypothetical substructure or motif composed of 3 to 8 residues that users can define using the interface provided.	• Selected proteomes by Alphafold • A manually curated PDB subset consisting mainly of proteins with non-redundant folds; • Specific requests from users that are also made accessible to other users.
RNA	NASSAM	Search for the presence of a 3D substructure composed of base arrangements in an structure containing RNA chains.	Structure coordinate file (*.pdb, *.cif) containing RNA chains.	• RNA base arrangements from the Nucleic Acids Interaction Library (37). • RNA base arrangements from NCIR (38) • Other manually curated motifs (20).
	COGNAC	Search for clusters of RNA bases that are interconnected by at least one hydrogen bond.	Structure coordinate file (*.pdb) containing RNA chains and base connection pattern options of 2 to 6 bases. An option to upload two files for comparisons is available.	• PDB structures containing RNA chains (with resolution of 3.5A or higher). • A user provided PDB formatted structure containing RNA chains as a comparison structure.

## RESULTS AND DISCUSSION

Our experience in operating five separate substructure searching servers since 2012 revealed that at times there were mismatches in the program selected by the users and the searches that they intended to execute. This motivated us to develop a single one-stop interface that enables users to annotate specific substructures in protein and RNA structure coordinate data without needing to know the specific operations of each tool. The substructure searching methods that we report here have been useful in the discovery of several structural motifs or for assigning functions when no fold similarity could be detected. The ability to search for similar sites in structures that have no detectable sequence similarity is useful in the identification of conserved mechanisms or functions among highly divergent members of a protein family or convergently evolved sites. One recent analysis that utilized the ASSAM server for substructure searching in such a way was the discovery of a four aspartates arrangement (4D motif) that is involved in binding a divalent metal ion to stabilize the acetylcholinesterase of *Torpedo californica* (30). Another example had used ASSAM to identify a 3D motif that could be regarded as a fingerprint for the fold of a protein superfamily (31).

The use of substructure searching has also proven useful for applications such as drug repositioning. Protein-ligand binding interfaces to approved drug compounds found in DrugBank (32) had been used as ASSAM queries to build a database of sites that are similar to known drug binding sites that are found in unrelated protein structures thus presenting the potential for such compounds to be repositioned to new targets (17). The potential drug repositioning sites were then made available as a database that could then be searched using the SPRITE search engine. The same principle that allows a compound to be repositioned for therapeutic outcomes is also applicable for causing toxicity or other side effects due to off-target binding. Therefore, the identification of such sites in human proteins could also provide insights into potential side-effects (18). This can be a useful tool to investigate the reported side-effects to known drugs and can be a useful aid for future clinical trials programs not only in the context of drug repurposing but also for the development of new drugs.

With the protein structure models computed using AlphaFold2 (6) for whole proteomes of model organisms and other medically important organisms now available (7), there is a need to identify whether known 3D motifs are present in a predicted structure that has no sequence or fold similarity to examples available in the PDB. Such proteins are often predicted as hypothetical proteins from the genome sequence data. There may also be structures that share a similar fold but yet contain a 3D motif that is different from that found in other PDB examples. Substructural similarities such as these could provide clues regarding a specific direction to take for assays that can validate the functional mechanism and ultimately assign the correct function to the protein. As previously mentioned, the ability to search human protein structure models for amino acid side chain arrangements that are similar to known drug binding sites is an important capability that can also be useful to find off-target sites similar to known drug binding sites that can in turn provide insights into potential toxicity or side effects.

There are a number of available web servers that can carry out local structural similarity searches, however, only GrAfSS is able to search and annotate for substructural similarities in both proteins and RNA (Table 2). We carried out a comparison of web servers that are similarly intended to GrAfSS using parameters classified under types of input files or query formats, the databases that they use if relevant, the objective of the search and the types of output or visualization options available (Table 2). This comparison clearly shows that no one resource is able to fulfil all the functions a diverse group of users might require. The currently available tools complement each other by filling in the gaps albeit with some core overlaps. GrAfSS, being a new service that integrates the functionalities of five different search programs, is able to provide a unique set of search functions that are not available via the other tools, including the use of side chain superpositions as opposed to C-alpha matches previously mentioned.

**Table 2.** Comparison of the different web servers that can up to an extent be used for the detection of substructural similarities and 3D motifs in the structures of proteins and RNA; features only found in the GrAfSS webserver are marked with an*

	Available Comparable Webservers													
	Ef-Seek (39)	GrAfSS	MultiBind (40)	ProFunc (41)	PDBeMotif (42)	ProBis (43)	R3D-BLAST (44)	RAG-3D (45)	RASMOT-3D Pro (11)	RCLICK (46)	SA-Mot (47)	SETTER (48)	SuMo (49)	WebFR3D (13)
**Input query format**														
PDB ID		**✓**	✓	✓	✓	✓	✓	✓		✓	✓	✓	✓	✓
Protein structure coordinate file in PDB format	✓	**✓**		✓	✓	✓			✓		✓		✓	
Protein structure coordinate file in mmCIF format*		**✓**												
User-defined query of conceptual amino acid arrangements / nucleic acid arrangement / interaction		**✓**	✓		✓				✓					✓
Structure coordinate file containing RNA chain(s) in PDB format		**✓**					✓	✓		✓		✓		
Structure coordinate file containing RNA chain(s) in mmCIF format*		**✓**												
**Databases searched against**														
Representatives of the PDB		**✓**		✓	✓		✓	✓		✓				✓
3D arrangements (ie. motifs, functional site, ligand binding site)	✓	**✓**	✓	✓	✓	✓			✓		✓		✓	✓
AlphaFold structures at EBI*		**✓**												
**Searches / Predicts for**														
Homologous structures (fold similarity)				✓	✓									
Local structural similarity	✓	**✓**	✓	✓	✓	✓	✓	✓	✓	✓	✓	✓	✓	
Pairwise structural similarity				✓		✓						✓		
Catalytic sites		**✓**		✓	✓									
Ligand binding sites	✓	**✓**	✓	✓	✓	✓							✓	
DNA/RNA-binding sites		**✓**		✓	✓	✓								
Protein-protein interfaces		**✓**			✓	✓								
Various 3D motifs	✓	**✓**	✓	✓	✓	✓		✓	✓		✓		✓	✓
Similar 3D arrangements to known drug binding sites*		**✓**												
**Output type / format**														
List of predicted 3D motifs / substructure ranked by structural similarity scores (ie. RMSD)	✓	**✓**		✓		✓	✓	✓	✓	✓	✓			
Direct molecular visualization of results - 3D motifs / substructure enabled	✓	**✓**	✓	✓	✓	✓	✓	✓	✓	✓	✓	✓	✓	✓
Downloadable output files	✓	**✓**	✓			✓	✓	✓	✓	✓		✓	✓	✓

### Case studies

**Figure 3.**
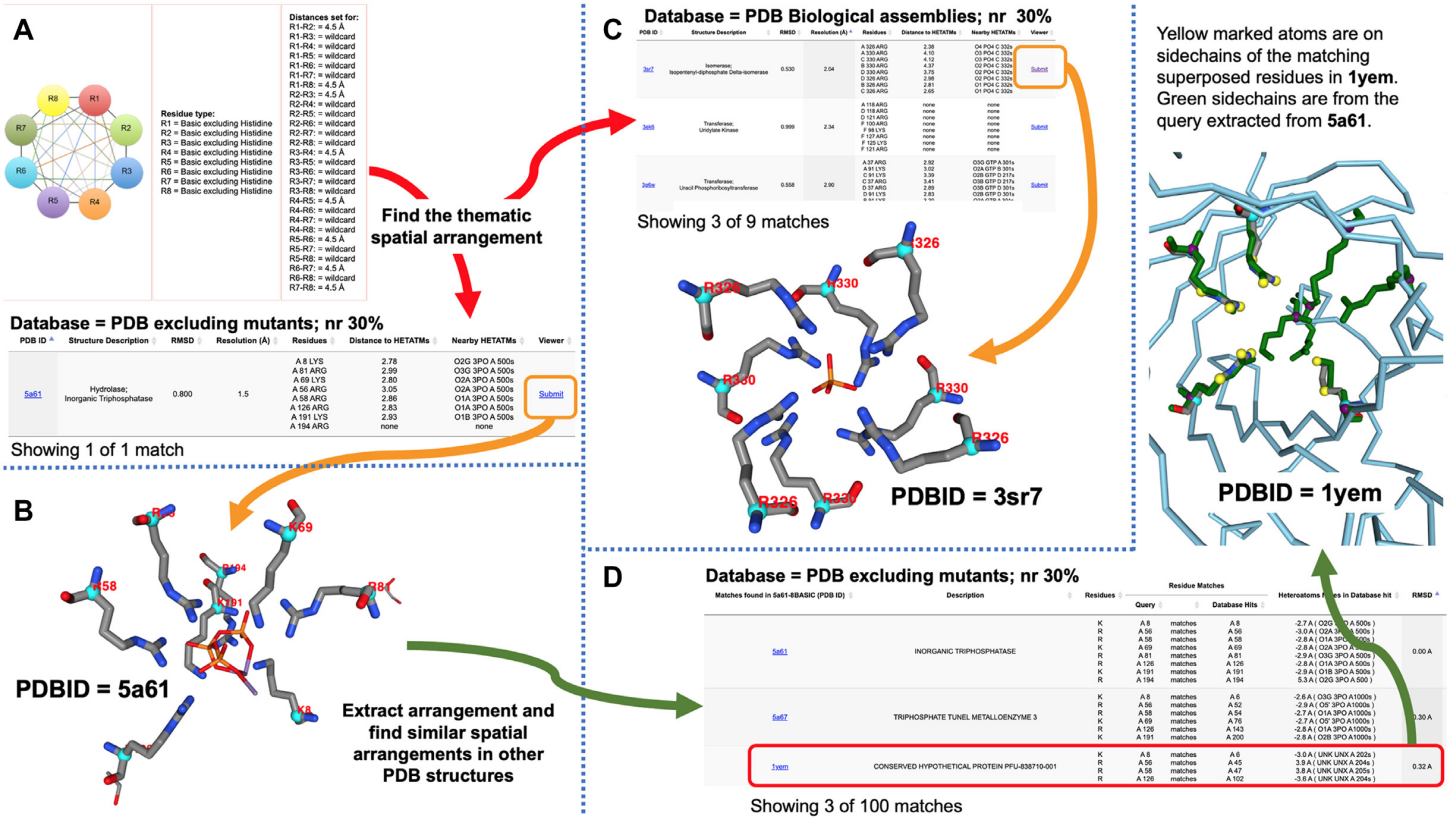
Example of a search process that a GrAfSS user can carry out to (**A**) explore a thematic spatial formation by providing a conceptual amino acid arrangement and searching: (**B**) a database of non-redundant PDB structures or (**C**) a database of biological assemblies from the PDB. (**D**) The results of the search can then be further investigated by providing the specific arrangement as a query to determine whether there are other representative structures in the PDB that also contain a similar arrangement. All search results can then be visualized using the embedded NGL viewer as presented in (B), (C) and (D).

The results of all GrAfSS searches are presented in tables as a web page (Figure 3). Depending on the types of searches carried out, some of the displayed outputs provide additional features for sorting or filtering results using the various pre-set buttons or pull-down menus such as, but not limited to options for: sorting the searches by hits that are in the proximity of heteroatoms, sorting according to the root mean square deviation (RMSD) of the superimposed substructures and filtering whether hits occur on the same chain or are composed of different chains. The specific case studies for each individual webserver have been presented previously in their respective associated publications. Here, we focus on how the different programs can be used together since such an example has not been previously reported in the literature.

Our example starts with a search to investigate for the presence of a spatial theme consisting of eight basic amino acid residues excluding histidine that are present in close 3D proximity (Figure 3A). This query is presented to search a database of non-redundant structures (cutoff at a maximum of 30% sequence identity) from the PDB that excludes any mutant structures (Figure 3B) and a database of biological assemblies also from the PDB (Figure 3C). The first search retrieved only a single example (Figure 3A; PDB ID = 5a61) of a KRRKRRKR arrangement that is part of a phosphate binding tunnel in the structure of an inorganic triphosphatase from *E. coli* K-12 (33). The search using the biological assemblies database retrieved one example of a *Streptococcus mutans* isopentenyl pyrophosphate isomerase (PDB ID = 3sr7) with an eight arginine (8R) arrangement that is at the interface of four different chains where each chain contributes two arginines to the arrangement (Figure 3C). Both the 8R and KRRKRRKR hits appear to be part of a positively charged spatial theme that in many examples are bound to phosphates.

The unique KRRKRRKR arrangement was then extracted as a PDB coordinate file and used as a query to identify whether other proteins in the 30% non-redundant PDB dataset contain an arrangement that is similar to it. This search retrieved 100 matches, one of which is a KRRR arrangement in a conserved hypothetical protein from *Pyrococcus furiosus* (PDB ID = 1yem) that remains annotated as having uncharacterized function and is unpublished. This structure appears to have been solved before other examples of such proteins were available; however superimpositions of the query to the match revealed that the arrangement could also potentially function as a site for binding phosphates (Figure 3D). This simple series of searches have demonstrated how a potentially novel motif can still be discovered and the functional context provided for decades old data as well as more recent entries in the PDB. We also show how GrAfSS can facilitate the discovery of not only novel motifs but also spatial themes that can provide insights for further functional characterization to be carried out.

## CONCLUSION AND OUTLOOK

The increasing availability of structure coordinate data is being supplemented by an even faster rate of generation for protein structure models that can be accurately predicted from available genome sequences. This huge leap in data availability requires tools that can complement fold similarity searching tools, especially when both sequence and fold similarity searches return dead ends or are inconclusive. The GrAfSS webserver fills this gap in being able to find and annotate known structural arrangements or 3D motifs, as well as aid in the discovery of novel 3D motifs in proteins as well as structures that contain RNA chains. Such substructure similarity searching has proven to be of great utility for investigating conserved functions at the atomic level and can also play a crucial role in providing a more mechanistic understanding of efficacy and toxicity during the development and clinical trials stages of new and repurposed drugs.
